# Physician Satisfaction in a Cancer Prevention Program for Low-Income Women in Nevada

**DOI:** 10.1100/tsw.2007.50

**Published:** 2007-02-09

**Authors:** Christopher R. Cochran, Emmanuel C. Gorospe

**Affiliations:** Department of Health Care Administration & Policy, School of Public Health, University of Nevada Las Vegas, Nevada, NV, USA

**Keywords:** cancer prevention, outpatient health services, indigent care, delivery of health care, physicians, Nevada

## Abstract

Physicians and health care organizations that provide services to low-income patients are valuable partners in improving health care access for the uninsured and medically underserved. In this pilot study, we explored physicians' needs and factors for satisfaction in the Women's Health Connection (WHC), a breast and cervical cancer-screening program for low-income women in Nevada. Of the 126 physicians in the WHC program, 50 physicians completed a needs-and-satisfaction questionnaire. Survey data were subjected to factor analysis using Varimax rotation. The results yielded three components, which accounted for 65% of the variance. The three components or dimensions for physician satisfaction were: (1) appropriate administrative support and documentation, (2) availability of support for medical management, and (3) timeliness of diagnostic reports. Amount of reimbursement was not a significant factor. The respondents serving in this cancer prevention program for low-income women were satisfied in their involvement in the program. Further attention should be given on the identified issues for satisfaction among physicians, which could lead to quality improvement and serve as a model for other programs that serve low-income patients in cancer prevention.

### INTRODUCTION

Physicians and health care organizations that provide services to low-income patients are valuable partners in improving health care access to the uninsured and medically underserved. In an era where health care providers are less inclined to participate in government-sponsored programs, it is important for these programs to implement policies that will enhance health care providers' support and participation. There are limited studies that have evaluated physicians' needs and satisfaction with community-based health care programs for the uninsured population in the U.S.[1,2]. Patient relationships, time spent with patients, working hours, and adequacy of resources have all been found to influence physician satisfaction[3]. A study[4] of primary care physicians in Medicaid, a national insurance program for indigent Americans, found that physician participation was positively associated with increased reimbursement rates, availability of a larger patient base, and physicians' perception of autonomy in clinical practice. In contrast, physician satisfaction was negatively associated with higher practice and operational costs, competition for paying patients, and differences between the marginal revenue derived from private paying patients compared to Medicaid beneficiaries.

In this article, we present physicians' needs and measures of satisfaction for services provided by the Women's Health Connection (WHC) program, the state of Nevada's breast and cervical cancer prevention program for low-income women. The program is funded through a grant from the Centers for Disease Control and Prevention (CDC) as part of the National Breast and Cervical Cancer Early Detection Program, which began providing breast and cervical cancer screening services for women ages 40 to 64 years old in Nevada in 1997. The program financially supports cervical and breast cancer screening services which include clinic visits, pelvic exams, Pap smears, clinical breast exams, and mammography. Reimbursement rates for patient services were established at state Medicaid levels. To assure that eligible women throughout the state can participate in the program, the WHC program has been recruiting physicians in private practice, including community, and hospital-based primary care programs. However, the major phase of physician recruitment was from the program's establishment in 1997 up to 2003. This same span of time coincides with our selection criteria for inviting WHC-participating physicians in this survey.

Nevada lags behind most states with established health care indicators. It has the 15$^{th}$ highest overall rate in cancer mortality in the U.S.[5]. With a cumulative population growth rate of 81% since 1990[6], the state has one of the largest population of uninsured adults[7]. It ranks 48$^{th}$ in the number of physicians per 100,000 population among the 50 U.S. states[8]. Moreover, the state has one of the highest tobacco and alcohol consumption rates and other unhealthy lifestyle behaviors that may contribute to cancer development[9]. The combination of the state's higher health risks and a lower than average number of health care providers requires programs such as the WHC program to meet provider needs and assure their continued participation.

By understanding the needs of physicians participating in federally funded community health care programs such as the WHC program, government programs can provide strategies to improve the quality of services for indigent women and make breast and cervical cancer prevention programs popular and more effective for both physicians and patients. Assessment of physicians' satisfaction can provide information for program evaluation and quality improvement. The private health care industry has recognized that continually measuring physician satisfaction is critical for its organizational success. Physician satisfaction helps managed care networks to retain physician staff and to recruit new members[10].

Previous studies have shown physicians satisfaction with health care programs to be associated with better patient care and program outcome[11,12]. Moreover, satisfied physician-providers have contributed to better patient compliance and satisfaction[13,14]. Improving physician satisfaction may improve the providers' willingness to participate and to continue supporting government-funded community-based health programs. Maintaining their services is crucial in a state like Nevada, where medical services and manpower are limited.

### METHODS

#### Instrument

A two-page survey instrument was developed based on information obtained from the WHC Program Staff, CDC performance measures, other breast and cervical cancer survey instruments, and literature review. A cover letter was included explaining the need to obtain physicians' needs and satisfaction assessment in order to improve the quality of services for WHC patients. Physician-providers were asked to complete a questionnaire, assisted by a small paragraph of instruction at the beginning of the page. A

portion of the questionnaire was allotted for informed consent. The approval to carry out this research was given by the State Health Division of Nevada.

We asked WHC-participating physicians about the types of services their office provided such as clinical breast exams, Pap smears, and pelvic exams. We also collected demographic data regarding their office practice, clinic location, number of physicians in the practice, and estimated proportion of WHC patients who are seen at their offices in comparison to their total number of patients.

On the second part of the questionnaire, we asked WHC-participating physicians to rate their level of agreement using a 5-point Likert scale starting with 1 as “strongly disagree” going up to 5 as “strongly agree”. There were 5 Likert-type questions in the survey on the possible needs of WHC-participating physicians. These included the need of (1) designating who should follow up WHC patients, (2) communicating with WHC patients regarding the program benefits, (3) assuring patients’ access to the participating clinics, (4) the need to be familiar with WHC documentation requirements and (5) the need for regular feedback from the WHC program regarding the clinic’s compliance to the program guidelines.

Using the same Likert scale, we assessed physician satisfaction with questions regarding whether or not their office receives (1) Pap smear and (2) mammography reports on time, (3) whether they are satisfied with the communication with the WHC program staff (i.e., ease of contacting and requesting information from WHC staff), (4) the amount of technical support they receive (i.e., availability of clear procedures and guidance for implementing the program), (5) provision of training for implementing the program, (6) amount of required documentation for reimbursement, (7) ease of completing the WHC forms, (8) amount of WHC reimbursement, (9) availability of follow up information on the referrals they send out for WHC patients and (10) awareness of other resources such as Medicaid coverage which can be availed for the benefit of participating patients. Finally, we asked about the physicians’ overall satisfaction and perceptions of the program with the questions, “Overall, the program is very helpful in providing support services” and “Overall, the program provides valuable services to patients under our care.”

### Participants

A list of physician was obtained from the Nevada State Health Division. We identified a total of 126 participating physicians throughout Nevada who had provided services for the WHC program between the years 1997-2003. This group included physicians in private practice, community based health care clinics, and community hospitals. We only included physicians who had participated in the program at some point between the years 1997-2003. This time frame would have allowed enough time for the physicians to be familiar with the WHC program. Although this contention was not assessed in this survey and could be identified as a limitation, the rationale for selecting such cohort of providers is acceptable, considering that physicians who have been working with the WHC for several years or who had served more patients in the program, and thus more paper work experience may reasonably have a more in depth assessment.

### Survey

The questionnaires were first mailed in June 2003, which yielded 35 responses. Follow-up surveys were faxed to physicians who had not responded to the first mailing. Physicians who have not responded thereafter were reminded by a telephone call. Thus, a total of two follow-up attempts (i.e., 1 fax message & 1 telephone call) were done prior to ending the survey. A total of 50 completed surveys were included in the study.

### Statistical Analysis

Surveys were analyzed using SPSS® version 13 statistical analysis software. Frequency rates, means, and standard deviations were obtained on relevant questions. We subjected the data to factor analysis to uncover latent variables, merge the different responses, and produce the principal factors that could explain the observed variance in the satisfaction measures among the participants. Prior to factor analysis, we subjected our data to the Kaiser-Meyer-Olin test to measure the adequacy of our sample for factor analysis and the Bartlett's test to check for the validity of our correlation matrix. For the questions pertaining to satisfaction with the program, components were extracted using iterative principal axis factoring. The Kaiser-Guttman and Scree tests were conducted to determine the number of components. These tests yielded three components and were subjected to Varimax rotation. Only factors with heavy loading (> 0.6) were included in the interpretation of component contents.

## RESULTS

The majority (54%, n=27) of the respondents were from Southern Nevada that includes the Las Vegas metropolitan area, while Northern Nevada, which includes the Reno metropolitan area, accounted for 16% (n=8) of the responses. Other rural Nevada counties had a combined representation of 30% (n=15). This represents the majority of Nevada's population and health care resources, which are located in the Las Vegas and Reno metropolitan areas. Among the providers surveyed, 62% (n=31) were operated by solo practice physicians. Only 10% (n=5) had 4 or more physicians working as a group practice clinic.

Physicians were asked about the types of services they provide. There were 72% (n=36) physicians who offered pelvic exams, 74% (n= 37) provided Pap smear collection, and 80% (n=40) performed clinical breast examination. Most physician-providers (60%, n=30) sent out Pap smear to outside pathology laboratories and 78% (n=39) also had to refer patients to an outside imaging facility for mammography.

In Figures 1 and 2, the percentages of WHC patients who receive cervical and breast exams respectively are compared to the total number of women who receive similar screening tests in the physicians' clinics. For example in Figure 1, WHC program patients accounted for 21-30% of the total number of cervical cancer screens in 33% of the clinics; 31-50% of all cervical cancer screens were WHC patients in 26% of the clinics; 5-10% were WHC patients in 17% of the clinics; 11-20% were WHC patients in 13% of the clinics; and less than 5% were WHC patients in 11% of the clinics.

Figure 2 illustrates a similar number. For example, WHC program patients accounted for 21-30% of the total number of cervical cancer screens in 32% of the clinics; 31-50% of all cervical cancer screens were WHC patients in 29% of the clinics; 11-20% were WHC patients in 19% of the clinics; 5-10% were WHC patients in 10% of the clinics; and less than 5% were WHC patients in 10% of the clinics.

Table 1 summarizes physicians' needs by indicating their level of agreement with a series of questions on a 5-point Likert scale. A mean score for each measure was calculated together with the frequency distribution. A higher mean score indicates that the respondent was more likely to agree with the statement for the identified item. Results from Table 1 indicate that there is a slightly increased need for WHC program staff to assist physicians' clinics in following-up WHC patients, communicating with patients regarding the program, and providing regular feedback to participating clinics.

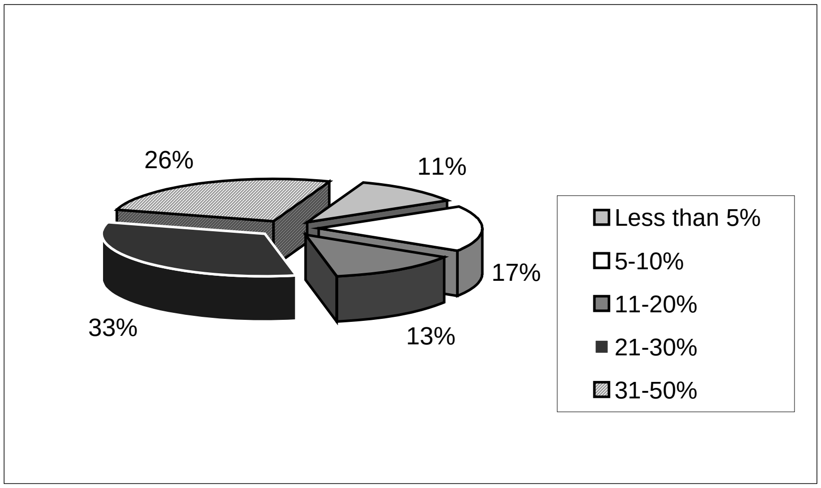

**Figure 1.** Percent of cervical exams provided to WHC patients compared to general patient population in Nevada WHC-participating clinics

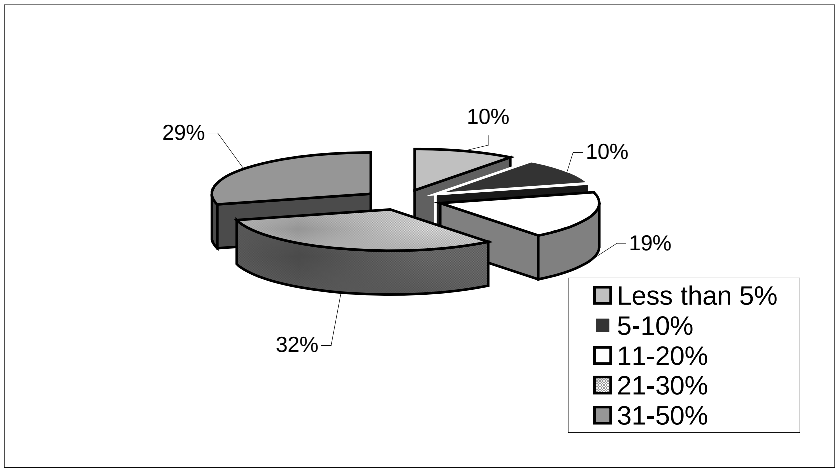

**Figure 2.** Percent of clinical breast exams provided to WHC patients compared to general patient population in Nevada WHC-participating clinics

Overall, 82% of physicians agreed (Likert mean of 4.38± 0.98) that the WHC program provided valuable services to their patients. However, only 70% of physicians reported satisfaction (3.92± 1.05) with the WHC program (data not shown). To assess the principal factors for physicians' satisfaction in the WHC program, we performed a factor analysis on the 10 questions regarding satisfaction. With 50 respondents, the Kaiser-Meyer-Olin measure of sampling adequacy was 0.69, suggesting satisfactoriness of our sample for the statistical test. Bartlett's test of sphericity was also <0.05. Each item's mean and standard deviation may be found on Table 2.

**TABLE 1**

**Assessment of Physicians' Needs in the Nevada WHC Program**

| Needs | Mean & Standard Deviation | Strongly Agree | Agree | Neither Agree nor Disagree | Disagree | Strongly Disagree |
|---|---|---|---|---|---|---|
| WHC case managers should be responsible for follow-up of WHC patients. | 3.84 (1.23) | 40.80 | 22.40 | 22.40 | 8.20 | 6.10 |
| Communicating with WHC patients is not a problem in our practice/clinic. | 3.90 (1.21) | 42.90 | 22.40 | 22.40 | 6.10 | 6.10 |
| Access to our facility is not a problem for WHC patients. | 4.47 (1.08) | 71.40 | 18.40 | 2.00 | 2.00 | 6.10 |
| This practice/clinic is familiar with all reporting requirements for the WHC. | 3.98 (1.19) | 46.00 | 22.00 | 22.00 | 4.00 | 6.00 |
| Clinic would benefit from regular reports regarding program compliance. | 3.98 (1.09) | 42.90 | 22.40 | 28.60 | 2.00 | 4.10 |

n= 50 respondents

Likert scale: 1=strongly disagree, 5= strongly agree

On a 5-point Likert scale (1 represented dissatisfaction while 5 represented strong satisfaction), the highest mean was 4.22 on the item regarding reporting of Pap smear results. Overall, the physician satisfaction grand mean was 34.92. In this survey, the highest possible score for physician satisfaction was 50. This suggested that physicians were moderately satisfied with the program.

The Kaiser-Guttman and Scree tests indicated that there are three components from the 10 item-questions on physician satisfaction. All three components accounted for 65% of the total variance. The three components or dimensions in order of importance based on Eigenvalues were satisfaction with (1) appropriate administrative support and documentation, (2) availability of support for medical management, and (3) timeliness of diagnostic reports. (See Table 3)

Five items were highly loaded on the first component: appropriate administrative support and documentation. These included: (a) satisfaction with communication between WHC program and clinic staff, (b) level of support from the program, (c) provision of training on reporting requirements, (d) reasonable documentation requirements, and (e) ease of completing WHC forms. There were two items loaded on the second component which pertains to the (a) awareness of Medicaid coverage/eligibility for WHC patients needing further diagnosis and treatment and (b) communication with other non-WHC participating physicians who may be consulted for specialist's opinion and services. Reimbursement issues did not load strongly on any of the components identified.

**TABLE 2**
**Means and Standard Deviation of Items for Physician Satisfaction in the Nevada WHC Program**

| Items | Mean & Standard Deviation | Strongly Agree | Agree | Neither Agree nor Disagree | Disagree | Strongly Disagree |
|---|---|---|---|---|---|---|
| Timeliness of Pap smear reporting | 4.22 (1.34) | 64.00 | 20.00 | 2.00 | 2.00 | 12.00 |
| Timeliness of Mammography reporting | 3.88 (1.41) | 48.00 | 24.00 | 8.00 | 8.00 | 12.00 |
| Communication with non-WHC physicians for referrals | 3.02 (1.38) | 22.00 | 12.00 | 28.00 | 22.00 | 16.00 |
| Ease of completing WHC forms | 3.84 (1.02) | 32.00 | 30.00 | 30.00 | 6.00 | 2.00 |
| Reasonable document requirements | 3.43 (1.22) | 22.40 | 26.50 | 32.70 | 8.20 | 10.20 |
| Provision of training on reporting requirements | 3.29 (1.23) | 22.4 | 18.4 | 30.6 | 22.4 | 6.10 |
| Awareness of Medicaid coverage for diagnosed patients | 3.36 (1.66) | 42.00 | 12.00 | 8.00 | 16.00 | 22.00 |
| Communication between WHC program and clinic staff | 3.50 (1.09) | 22.00 | 24.00 | 42.00 | 6.00 | 6.00 |
| Level of support from WHC program staff | 3.92 (1.05) | 34.00 | 36.00 | 22.00 | 4.00 | 4.00 |
| WHC Reimbursement contributing significantly to operating budget | 2.47 (1.31) | 10.20 | 10.20 | 26.50 | 22.40 | 30.60 |

n = 50 respondents

Likert scale: 1=strongly disagree, 5= strongly agree

### DISCUSSION

Our results suggest a discrepancy between the physicians' level of satisfaction and perception of the program's benefit to their patients. To further analyze this issue, we investigated the factors that contribute to physician satisfaction. The component concerning the level of administrative support and ease of documentation requirements in the program accounted for the most variance among the factors measuring physician satisfaction. This finding is parallel to the physician's needs assessment in Table 1 which indicates the need to be familiar with reporting requirements and program compliance as among their priority needs.

For community-based health programs such as the WHC, an important implication is that physician-providers will be more satisfied with their participation if they feel that they are getting sufficient support from the state agency that oversees the program. In addition, training was also a significant factor.

Training may familiarize physicians and their staff on the program's reporting and documentation which may improve program utilization.

**TABLE 3**
**Physician Satisfaction Pattern Matrix in the Nevada WHC Program**

| Items | Component | | |
|---|---|---|---|
| | 1 | 2 | 3 |
| Communication between WHC program and clinic staff | **0.86** | 0.26 | -.031 |
| Level of support from WHC program staff | **0.86** | 0.20 | 0.18 |
| Provision of training on reporting requirements | **0.70** | 0.15 | -0.04 |
| Reasonable documentation requirements | **0.66** | -0.20 | 0.33 |
| Ease of completing WHC forms | **0.61** | -0.04 | 0.40 |
| Awareness of Medicaid coverage for diagnosed patients | 0.10 | **0.88** | 0.04 |
| Communication with non-WHC physicians for referrals | 0.27 | **0.73** | 0.10 |
| Timeliness of Pap smear reporting | 0.17 | 0.05 | **0.78** |
| Timeliness of Mammography reporting | -0.08 | 0.45 | **0.78** |
| WHC Reimbursement contributing significantly to operating budget | -0.19 | 0.36 | -0.45 |
| Eigenvalue | 3.52 | 1.60 | 1.38 |
| Percent of Variance Explained | 35.21 | 16.05 | 13.78 |

Extraction Method: Principal Component Analysis.
Rotation Method: Varimax with Kaiser Normalization.
Rotation converged in 6 iterations.
n= 50 respondents

Efficient and simple documentation was also an important contributor to physicians' satisfaction. Our results support initial reports[15] suggesting increased documentation to negatively affect physician satisfaction. Focus group discussions and anecdotal reports from physicians participating in similar Medicaid programs mention cumbersome documentation as a source of dissatisfaction. Increased paperwork creates extra burden to clinic staff which may require reorganization of staff duties, hiring of additional employees, and bigger operational expenses[16].

Overall, the program did not provide significant contribution to the operating budget of participating clinics. In the Nevada WHC program, reimbursement does not significantly contribute to physicians' satisfaction contrary to earlier reports suggesting financial reimbursement as a factor for general job satisfaction and participation in similar Medicaid programs among physicians[17-20]. Our results support current findings suggesting financial reimbursement as becoming more unrelated to physician satisfaction[21]. We surmise that physicians may have lesser expectations for reimbursement especially from government programs intended for the care of indigent patients. Given the current trend for decreasing physician reimbursement in private and public health insurance programs, physicians might be looking for other non-monetary benefits for their participation and services.

The second dimension for physician satisfaction was the availability of medical support. Most physicians in the screening program were primary care physicians. Receiving follow-up information for patients referred for consultation with a specialist-physician assures continuation of care and availability of assistance for complicated cases that may need additional expert opinion or co-management. The awareness about patients' eligibility for Medicaid coverage in addition to the WHC benefits also contributed to physician satisfaction. This may assure physicians that their patients' medical care will not be hindered by insurance restrictions or lack thereof.

Lastly, as majority of WHC-participating physicians send out their pap smear for pathology consultation and referral for mammography to imaging facilities, timeliness of diagnostic reports comprised the third component of WHC-participating physicians' satisfaction. Under the WHC program, patients needing diagnostic tests are assisted in identifying and accessing WHC participating diagnostic and imaging laboratories. Prompt completion and reporting of these tests facilitates physicians' diagnosis and clinical management.

A major limitation of this study was the number of providers who responded. Only 50 providers out of 126 physicians responded despite follow-up telephone calls and faxing survey information. On average, mail surveys to physicians have a response rate of approximately 60%[22]. Another potential problem with the study is the lack of open-ended questions. While categorical questions are easier to answer and typically prompt a higher response rate, they also limit detailed input as to what physician-providers actually need to improve their satisfaction with the WHC program. Even though a comment section was provided at the end of the survey, few respondents were willing to share their opinion and the majority of them chose to leave the comment section blank.

### CONCLUSION

Overall, 82% of physicians agreed that the Nevada WHC program provided valuable services to their patients. This pilot study suggests that the following components: (1) appropriate administrative support and documentation, (2) availability of support for medical management, and (3) timeliness of diagnostic reports represent important dimensions of physician satisfaction with the WHC program in Nevada. As community-based programs do not provide significant financial gain to physicians' practices, future efforts should explore how to improve other issues affecting physicians' practice in order to promote and maintain physician participation in similar programs serving the low-income and medically underserved population.

### ACKNOWLEDGEMENTS

This study was funded through a grant from the Centers for Disease Control and Prevention (CDC) (Grant Number U55/CCU922006-02). The authors would like to thank Salome Kapella, MPA and Graciela Tena de Lara for their assistance in conducting the survey. An additional acknowledgement is made to the staff of the Women's Health Connection, Rocio Flores together with Deborah McBride and Andy DuMond of the Nevada State Health Division.
